# Expression of delta-like ligand 4 (Dll4) and markers of hypoxia in colon cancer

**DOI:** 10.1038/sj.bjc.6605368

**Published:** 2009-10-20

**Authors:** A M Jubb, H Turley, H C Moeller, G Steers, C Han, J-L Li, R Leek, E Y Tan, B Singh, N J Mortensen, I Noguera-Troise, F Pezzella, K C Gatter, G Thurston, S B Fox, A L Harris

**Affiliations:** 1 Nuffield Department of Clinical Laboratory Sciences, University of Oxford, John Radcliffe Hospital, Headington, Oxford OX3 9DS, UK; 2 Weatherall Institute of Molecular Medicine, University of Oxford, John Radcliffe Hospital, Headington, Oxford OX3 9DS, UK; 3 Nuffield Department of Surgery, University of Oxford, John Radcliffe Hospital, Headley Way, Headington, Oxford, OX3 9DU, UK; 4 Regeneron Pharmaceuticals, 777 Old Saw Mill River Road, Tarrytown, NY10591, USA; 5 Department of Pathology, Peter MacCallum Cancer Centre, St Andrew's Place, East Melbourne, Victoria 3002, Australia

**Keywords:** delta-like ligand 4, colon cancer, hypoxia, angiogenesis, survival

## Abstract

**Background:**

Delta-like ligand 4 (Dll4) is a Notch ligand that is upregulated by hypoxia and vascular endothelial growth factor-A (VEGF-A) and is reported to have a role in tumor angiogenesis. Evidence from xenograft studies suggests that inhibiting Dll4–Notch signalling may overcome resistance to anti-VEGF therapy. The aim of this study was to characterise the expression of Dll4 in colon cancer and to assess whether it is associated with markers of hypoxia and prognosis.

**Method:**

In all, 177 colon cancers were represented in tissue microarrays. Immunohistochemistry was performed using validated antibodies against Dll4, VEGF, hypoxia-inducible factor (HIF)-1*α*, HIF-2*α*, prolyl hydroxylase (PHD)1, PHD2, PHD3 and carbonic anhydrase 9 (CA9).

**Results:**

The expression of Dll4 was observed preferentially in the endothelium of 71% (125 out of 175) of colon cancers, but not in the endothelium adjacent to normal mucosa (none out of 107, *P*<0.0001). The expression of VEGF was significantly associated with HIF-2*α* (*P*<0.0001) and Dll4 (*P*=0.010). Only HIF-2*α* had a significant multivariate prognostic effect (hazard ratio 1.61, 95% confidence interval 1.01–2.57). Delta-like ligand 4 was also expressed by neoplastic cells, particularly neoplastic goblet cells.

**Conclusion:**

Endothelial expression of Dll4 is not a prognostic factor, but is significantly associated with VEGF. Assessing endothelial Dll4 expression may be critical in predicting response to anti-VEGF therapies.

Colorectal cancer is the second most common cause of cancer deaths worldwide, with an estimated 1 023 152 new cases and 528 978 related deaths in 2002(Parkin *et al*, 2005). The early growth of colorectal tumours requires angiogenesis (Goodlad *et al*, 2006; Korsisaari *et al*, 2007), the consequence of increased expression of pro-angiogenic factors (e.g., vascular endothelial growth factor-A (VEGF-A); Ferrara *et al*, 1991; Kim *et al*, 1993; Jubb *et al*, 2003; Korsisaari *et al*, 2007). The expression of VEGF in cancer is controlled by both oncogenic signalling (such as Wnt-signalling in colorectal cancer; Zhang *et al*, 2001) and hypoxia (Mizukami *et al*, 2004). Although there is redundancy among pro-angiogenic factors in advanced cancer (Relf *et al*, 1997; Hanrahan *et al*, 2003), many early cancers (Hanrahan *et al*, 2003; Jubb *et al*, 2003) and *in vivo* cancer models (Bergers *et al*, 1999; Hanrahan *et al*, 2003; Joyce *et al*, 2003; Goodlad *et al*, 2006; Korsisaari *et al*, 2007) are VEGF dependent. This observation has been exploited by the addition of an anti-VEGF monoclonal antibody (bevacizumab) to first-line chemotherapy in metastatic colorectal cancer, which prolonged the median overall survival (from 15.6 to 20.3 months, *P*=0.001; Hurwitz *et al*, 2004). Nevertheless, all patients in this trial eventually progressed after 2 years (Jubb *et al*, 2006b), and there are no valid predictors of the survival benefit afforded by bevacizumab in colorectal cancer (Ince *et al*, 2005; Jubb *et al*, 2006a; Grothey *et al*, 2008a, 2008b).

Additional therapeutic agents that disrupt functional tumour angiogenesis have been developed to target tumours that are inherently resistant to anti-VEGF therapy or become resistant during the course of therapy (Noguera-Troise *et al*, 2006; Ridgway *et al*, 2006; Pan *et al*, 2007; Caunt *et al*, 2008). Delta-like Ligand 4 (Dll4) is a ligand for Notch 1, 3 and 4 proteins, which is expressed by endothelial cells (Thurston *et al*, 2007; Indraccolo *et al*, 2009) and may be induced by VEGF and hypoxia, through hypoxia-inducible factor (HIF)-1*α* (Patel *et al*, 2005). In the retina, the interaction between Dll4 and Notch regulates endothelial sprouting and branching to form functional vascular beds (Hellstrom *et al*, 2007). Disruption of Dll4 signalling by overexpression or inhibition of Dll4 may impair tumour angiogenesis (Noguera-Troise *et al*, 2006; Ridgway *et al*, 2006), and blockade of Dll4–Notch signalling results in an increased density of non-functional vasculature and is associated with a reduction in the growth of human xenografts (Noguera-Troise *et al*, 2006; Ridgway *et al*, 2006). Indeed, certain xenografts that are resistant to anti-VEGF therapy are reported to be sensitive to anti-Dll4 (Noguera-Troise *et al*, 2006; Ridgway *et al*, 2006; Li *et al*, 2007), and combination treatment with anti-VEGF and anti-Dll4 has additive effects on tumour growth (Ridgway *et al*, 2006). In addition, human umbilical vein endothelial cells transfected with Dll4 downregulate the VEGF receptor 2 and co-receptor neuropilin-1, and show reduced proliferative and migratory responses to VEGF (Williams *et al*, 2006). Together, these data suggest that Dll4 may have a role in mediating resistance to anti-VEGF therapies.

The characterisation of Dll4 protein expression in human cancer is important for the rational design of clinical trials to test the safety and activity of anti-Dll4 therapy. Moreover, defining the context of Dll4 expression, in terms of known markers of hypoxia and angiogenesis, may identify subgroups of tumours with distinct clinical behaviour and response to treatment. The aims of this study were to characterise the *in situ* expression of Dll4 in colon cancer, to assess the association between Dll4 and established markers of hypoxia and angiogenesis and to determine the prognostic significance of these markers.

## Patients and methods

### Cell lines

Recombinant human Dll4 was cloned into a pcDNA3.1 vector (Invitrogen, Carlsbad, CA, USA). Vectors were transfected into human U87 cells with the Fugene 6 transfection reagent (Roche Applied Science, Indianapolis, IN, USA). Human umbilical vein endothelial cells were cultured *in vitro* in media with and without VEGF as previously described (Harrington *et al*, 2007). Cell pellets were then formalin fixed and paraffin embedded. Further details are available from the authors on request.

### Patients and tissue samples

Formalin-fixed and paraffin-embedded tissue blocks and corresponding pathology reports were retrospectively obtained for 177 sequential patients with colon adenocarcinomas (surgery was performed from 1997 to 2000 at the John Radcliffe Hospital, Oxford, UK). Sample size was determined by the availability of tissue with full clinicopathological data, survival follow-up and ethical approval for research. No patients received preoperative chemotherapy. Rectal cancers were excluded from this series to avoid the confounding effects that preoperative radiotherapy may have on the expression of hypoxic markers. The mean age at diagnosis was 71 years (range 17–92 years), 97 patients (55%) were male and 73 cancers (41%) were at or distal to the splenic flexure. In all, 13 cancers were in Dukes’ stage A (7%), 88 in stage B (50%), 55 in stage C (31%) and 21 in stage D (12%) at the time of surgery. The mean follow-up time was 5.6 years (range 1 month to 11.9 years). No patients received postoperative radiotherapy. No Dukes’ stage A patients, 38% (24 out of 64) of Dukes’ stage B patients, 58% (32 out of 55) of Dukes’ stage C patients and 52% (11 out of 21) of Dukes’ stage D patients received 5-fluorouracil-based chemotherapy according to the local protocols (adjuvant regimen: six 5-day courses of 370 mg m^−2^ 5-fluorouracil and 25 mg L-folinic acid as an intravenous bolus every 4 weeks). Follow-up data were correct as of January 2009, with a median follow-up time of 5 years and 7 months. Tissue microarrays were assembled as described previously (Bubendorf *et al*, 2001) with three replicate cores for each tumour and one core for each normal mucosal sample. An additional 12 whole sections of colorectal adenomas with adjacent adenocarcinoma were also collected (John Radcliffe Hospital) to further analyse the expression of Dll4 in the adenoma to adenocarcinoma sequence. Approval was obtained for the use of all human tissue from the local research ethics committee (C02.216).

### *In situ* hybridisation

Isotopic *in situ* hybridisation for Dll4 was performed using previously described methods (Poulsom *et al*, 1998). The probe used was a 741-bp cloned fragment of human Dll4 (position 1775 to 2516 bp, relative to the adenosine of the start codon ATG; Patel *et al*, 2006). *In situ* hybridisation for *β*-actin was used as a positive control for mRNA integrity.

### Immunohistochemistry

Immunohistochemistry for VEGF (clone VG1; Turley *et al*, 1998; Boddy *et al*, 2005), HIF-1*α* (clone 54, BD Transduction Laboratories, San Jose, CA, USA) (Wiesener *et al*, 2001), HIF-2*α* (clone EP190/E10; Talks *et al*, 2000; Boddy *et al*, 2005), prolyl hydroxylase 1 (PHD1) (clone 112/E8), PHD2 (clone 366G/76/3), PHD3 (clone EG188e/E6) (Appelhoff *et al*, 2004; Boddy *et al*, 2005; Soilleux *et al*, 2005) and carbonic anhydrase 9 (CA9) (clone M75, a gift from Professors S Pastorekova and J Pastorek, Institute of Virology, Slovak Academy of Sciences, Bratislava, Slovak Republic) (Pastorekova *et al*, 1992; Loncaster *et al*, 2001) was performed using antibodies as previously described. Validation of all antibodies has been previously undertaken and published by our group using cells transfected with the relevant targets (see references listed above). Unless otherwise stated, all antibodies were made in the Nuffield Department of Clinical Laboratory Sciences at the University of Oxford, UK.

Immunohistochemistry for Dll4 was performed using an anti-Dll4 monoclonal antibody (the variable regions of this antibody are fully human and the Fc-domain is mouse; clone 242) that binds to the extracellular domain of human Dll4 (the epitope is in EGF-like domains 3–5 and generated in VelocImmune mice (Regeneron Pharmaceuticals, Inc., Tarrytown, NY, USA)). In brief, antigen retrieval was performed in target retrieval solution (Dako, Carpinteria, CA, USA) using a Decloaking Chamber (Biocare Medical, Concord, CA, USA). Sections were incubated for 16 h at 4 ^o^C with the primary antibody at 1 *μ*g ml^−1^. Bound antibody was labeled with Novolink polymer (Leica Microsystems, Bannockburn, IL, USA), visualised using 2,3-diaminobenzidine chromogen and counterstained with hematoxylin. Control sections of bladder cancer and normal kidney were included to assess the specificity of the antibody.

Staining for HIF-1*α* and HIF-2*α* was scored positive if expression was observed in any epithelial cell nuclei. Cores were scored positive for CA9 if membranous expression was observed in >10% of epithelial cells. Staining for VEGF, PHD1, PHD2 or PHD3 was scored positive if cytoplasmic expression was observed in >10% of epithelial cells. Cores were scored positive for Dll4 if expression was observed in any endothelial cells. In addition, Dll4 was scored in the neoplastic cells within TMA cores in which it was observed at high levels, that is, levels that approximated the intensity of Dll4 expression by endothelial cells. The highest intensity score among replicate cores was used as the score for each patient. Assays and scoring were performed blind to clinical and pathological data (withheld for analysis until all data were complete).

### Statistics

The *χ*^2^ test was used to evaluate associations between categorical variables. The Student's *t*-test was used to evaluate associations between continuous and categorical variables. The Benjamini and Hochberg false discovery rate controlling procedure was used to eliminate spurious statistical associations as a result of multiple testing (Benjamini and Hochberg, 1995). All survival analyses refer to overall survival times, in which death from any cause represents an event. Marker values were assessed as ordinal categorical data in survival analyses. Median overall survival times within each subgroup were estimated from Kaplan–Meier curves. Patients were censored in survival analyses according to the date last seen by a doctor. The log-rank (Mantel–Cox) test was used to assess the significance of univariate survival analyses. For multivariate survival models, a Cox regression analysis was used to identify independent prognostic factors. All statistical analyses were carried out using SPSS Statistics (version 16.0, SPSS, Chicago, IL, USA). The two-sided *P-*values of <0.05 were considered statistically significant. Cases with missing data were omitted from statistical analyses.

### Reporting recommendations for tumour marker prognostic studies (REMARK) criteria

The REMARK criteria of the National Cancer Institute were used in the design, analysis and interpretation of this research (McShane *et al*, 2005).

## Results

### Frequency and pattern of expression of hypoxia-regulated proteins

Analyses of tumours from the 177 patients yielded informative data on 155 to 177 patients. Results were not available for the remaining cases in certain assays because of the limited amounts of tissue represented in the tissue microarrays.

**Figure 1:**
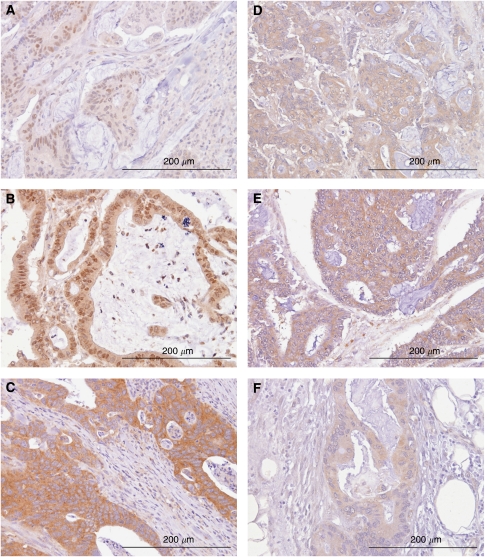
Representative examples of immunohistochemistry in colon cancer showing nuclear HIF-1*α* (**A**) and HIF-2*α* (**B**) expression, and cytoplasmic VEGF (**C**), PHD1 (**D**), PHD2 (**E**) and PHD3 (**F**) expression.

In all, 39% (60 out of 155) and 53% (85 out of 159) of colon cancers were positive for nuclear HIF-1*α* and HIF-2*α* expression, respectively; 21% of cases (31 out of 147) expressed both HIF-1*α* and HIF-2*α* and 51% of cases (75 out of 147) expressed either HIF-1*α* or HIF-2*α*. In addition, HIF-1*α* expression was only observed in cell nuclei when present (Figure 1A), as previously described (Jubb *et al*, 2004). The expression of HIF-2*α* by neoplastic cells was predominantly cytoplasmic, with nuclear expression in a proportion of cases (Figure 1B; Talks *et al*, 2000). The expression of HIF-2*α* was also observed in a proportion of infiltrating inflammatory cells as previously reported (Talks *et al*, 2000), although these data were not specifically recorded. The expression of VEGF was observed in the cytoplasm of neoplastic cells (88%, 149 out of 169), stromal fibroblasts, inflammatory cells, endothelial cells and the extracellular matrix (Figure 1C), as previously reported (Jubb *et al*, 2006a).

**Figure 2:**
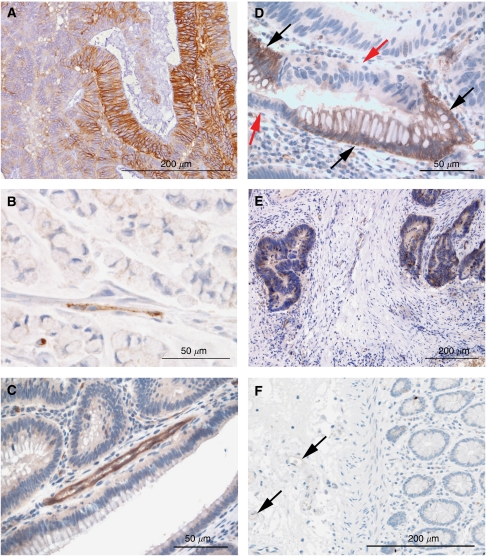
Representative examples of immunohistochemistry in colon cancer showing membranous CA9 adjacent necrosis (**A**) and endothelial Dll4 (**B**) expression. Immunohistochemistry for Dll4 shows membranous and cytoplasmic endothelial expression in a colon adenoma (**C**) and epithelial expression associated with goblet cell differentiation in a neoplastic crypt ((**D**) black arrows indicate Dll4-positive goblet cells, and red arrows indicate Dll4-negative non-goblet cells). Dll4 is also weakly expressed by neoplastic cells without goblet cell differentiation in a colon adenocarcinoma (**E**). Endothelial cells lining vessels (arrows) adjacent to normal colonic crypts did not express Dll4 by immunohistochemistry (**F**).

The PHD family of HIF-regulatory proteins is expressed in the cytoplasm of the majority of colon cancers (Figures 1D–F), similar to prostate cancer (Boddy *et al*, 2005). Prolyl hydroxylase 1 was present in 72% (116/162), PHD2 in 82% (135/164) and PHD3 in 70% (120/172) of colon cancers. Similarly, the majority of colon cancers show strong membranous expression of the HIF-1 target gene CA9 (67%, 110/165; Figure 2A), consistent with other series (Rasheed *et al*, 2009).

### Validation of the anti-Dll4 antibody

The human monoclonal antibody (clone 242) that specifically recognises the extracellular domain of human Dll4 did not stain U87 cells transiently transfected with empty vector (Supplementary Figure 1A), but showed membranous and cytoplasmic staining in U87 cells transiently transfected with the recombinant human Dll4 full-length gene (Supplementary Figure 1B). Human umbilical vein endothelial cells showed immunoreactivity for Dll4 that was greater in intensity when cells were treated with VEGF (Supplementary Figures 1C and D). Normal kidney did not express Dll4, but endothelial cells in renal cell carcinoma showed endothelial expression of Dll4, consistent with previous *in situ* hybridisation results (Supplementary Figures 1E and F) (Patel *et al*, 2005). Serial sections of bladder cancer showed endothelial colocalisation of Dll4 mRNA using *in situ* hybridisation (Patel *et al*, 2006), and protein using immunohistochemistry (Supplementary Figures 1D–F).

### Frequency and pattern of expression of Dll4

In 71% (125 out of 175) of colon cancer tissues analysed, Dll4 expression was observed in the cytoplasm of the endothelial cells lining vessels adjacent to cancer (Figure 2B). Membranous localisation of Dll4 was difficult to differentiate from cytoplasmic localisation because of the slender nature of endothelial cytoplasmic processes. The expression of Dll4 was only rarely observed in the endothelial lining of large vessels around or into which cancer cells had invaded. In the whole sections of colorectal adenomas with adjacent adenocarcinoma, Dll4 was observed at identical frequencies in the endothelium found in both the adenomas and adenocarcinomas (12 out of 12, Figure 2C, Supplementary Figures 2A–C). The endothelium associated with colonic adenomas and adenocarcinomas (i.e., within two low-power fields of the tumour cells) was positive for Dll4 in 60–100% of vessels (identified by CD34 immunoreactivity in serial sections, data not shown). Similar levels of endothelial Dll4 expression were observed in the centre of the tumour and at the invading edge. In all, 16% (28 out of 177) of colon cancers represented in the tissue microarrays expressed levels of Dll4 in the cytoplasm and membrane of neoplastic cells that approximated the intensity of expression observed in endothelium. In the whole sections, >10% of the malignant epithelial cells of the colorectal adenomas and adjacent adenocarcinomas expressed Dll4 in 8 of 12 cases (median 30%, range 10–60%). In regions of both colorectal adenomas and adenocarcinomas, high levels of Dll4 expression were observed in neoplastic epithelial cells with goblet cell differentiation (Figure 2D, Supplementary Figure 2D). (Goblet cells were defined morphologically.) Elsewhere, Dll4 was expressed at a lower level by neoplastic cells without goblet cell morphology (Figure 2E). Endothelial cells adjacent to normal colon mucosa distant from cancer did not express Dll4 protein (0 out of 107). Epithelial cells lining normal colonic crypts, irrespective of their morphology, did not express Dll4 (Figure 2F), although occasional cells in the superficial surface mucosa of normal colon stained weakly for Dll4 (data not shown). Specifically, normal colonic goblet cells did not express Dll4. The pattern of Dll4 expression by *in situ* hybridisation matched the immunohistochemistry results for the 12 colorectal adenomas with adjacent adenocarcinoma (Supplementary Figure 3).

### Associations between molecular and pathological variables

Hypoxia-inducible factor-1*α* and CA9 were not significantly associated with any other immunohistochemical markers in colon cancer (Table 1). In contrast, HIF-2*α* was significantly associated with VEGF, Dll4, PHD1, PHD2 and PHD3. Vascular endothelial growth factor was significantly associated with endothelial Dll4, PHD1, PHD2 and PHD3. Endothelial Dll4 was also significantly associated with PHD1 and PHD3. Prolyl hydroxylase (PHD)1, PHD2 and PHD3 were significantly associated with each other. In addition, endothelial expression of Dll4 was negatively associated with nuclear HIF-2*α* expression by tumour cells, but this association was not significant after correction for multiple testing. Delta-like ligand 4 expression by malignant epithelial cells was not associated with any clinicopathological or molecular variables (data not shown).

None of the immunohistochemical markers analysed in this study were associated with tumour stage, lymphatic invasion, vascular invasion, grade, sex or site after the false discovery rate controlling procedure was used to exclude spurious results (Table 2). Similarly, no immunohistochemical markers were associated with age using the Student's *t*-test (data not shown).

### Survival

**Figure 3:**
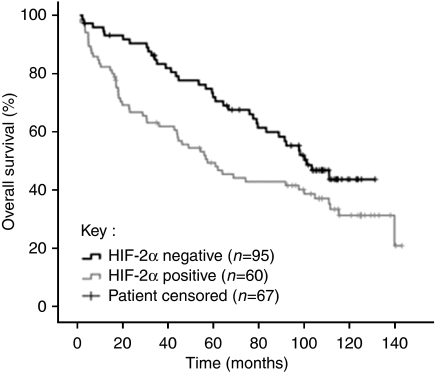
Kaplan–Meier survival curves for colon cancer patients subgrouped according to HIF-2*α* expression. The patients with cancers who are positive for HIF-2*α* have a significantly shorter median survival (57 months, 95% confidence interval 36–79 months) than patients with cancers who are negative for HIF-2*α* (101 months, 95% confidence interval 81–121 months), *P*=0.027.

In univariate analyses, only nuclear HIF-2*α* (*P*=0.027), pT stage (*P*=0.0003), pN stage (*P*<0.0001), M stage (*P*<0.0001), lymphatic invasion (*P*=0.007), vascular invasion (*P*=0.002), grade (*P*=0.028) and adjuvant chemotherapy (*P*=0.003) significantly correlated with overall survival (Table 3, Figure 3). (Note that the patients receiving adjuvant chemotherapy seem to have performed worse in overall survival analyses as they disproportionately represent advanced-stage cancers). The relationship between age and overall survival was assessed using Cox regression; it was not statistically significant, *P*=0.10. Patients with cancers that were positive for nuclear HIF-2*α* expression had a significantly shorter median survival (57 months, 95% confidence interval 36–79 months) than patients with cancers that were negative for nuclear HIF-2*α* expression (101 months, 95% confidence interval 81–121 months). In addition, HIF-2*α* showed statistical significance (*P*=0.044) in multivariate survival analyses (Table 4). The Cox regression multivariate survival model also included pT stage (*P*<0.05), pN stage (*P*=0.009), M stage (*P*<0.0001), lymphatic invasion (*P*=0.32), vascular invasion (*P*=0.48), grade (*P*=0.56) and adjuvant chemotherapy (*P*=0.56) (Table 4). The significance of statistical associations and survival analyses does not differ if the intensity of staining of the immunohistochemical markers was scored on a semiquantitative scale, as opposed to positive or negative (data not shown).

Endothelial Dll4 expression was not a statistically significant prognostic factor (Table 3). Although these are exploratory analyses, power calculations for Dll4 showed a statistical power of 0.882 and a hazard ratio of 0.5 for control subjects versus experimental subjects (based on a type 1 error probability of 0.05, an experimental group size of *n*=50, a control group size of *n*=125, an accrual time of 3 years, with 5-year follow-up and a median survival time of 6.94 years; Dupont and Plummer, 1990).

## Discussion

Spatial regulation of an appropriate level of Dll4 expression is important in the development of a functional vasculature during physiological and pathological angiogenesis (Duarte *et al*, 2004; Noguera-Troise *et al*, 2006; Ridgway *et al*, 2006). Moreover, Dll4 expression may influence the sensitivity of endothelial cells to anti-VEGF therapy, with potential implications for the use of bevacizumab and novel anti-Dll4 antibodies in colorectal cancer (Noguera-Troise *et al*, 2006; Ridgway *et al*, 2006; Williams *et al*, 2006; Li *et al*, 2007). This is the first study to perform a detailed evaluation of Dll4 expression in colon cancer, and to relate Dll4 expression to other known histological and prognostic markers.

In this series of colon cancers, Dll4 expression was observed in the cytoplasm of the endothelium lining small vessels of neoplastic, but not normal tissue. This is consistent with data from human xenografts (Noguera-Troise *et al*, 2006) and *in situ* hybridisation data for Dll4 in human bladder cancer (Patel *et al*, 2006) and human renal cell carcinoma (Mailhos *et al*, 2001; Patel *et al*, 2005). The lack of expression of Dll4 in the endothelial cells and epithelium of normal colonic crypts is consistent with the lack of VEGF expression by normal colorectal mucosa (Jubb *et al*, 2003) and the lack of intestinal toxicity and goblet cell hyperplasia observed in anti-Dll4-treated mice (Ridgway *et al*, 2006). These data suggest that strategies targeting Dll4–Notch signalling may selectively affect the immature developing endothelium associated with colon cancer, but not endothelium associated with normal colonic mucosa or normal colonic crypt epithelium.

The observation of Dll4 expression by neoplastic crypts and the association with goblet cell morphology in colon adenomas and adenocarcinomas is novel. However, a report by van Es *et al* (2005) claims that inhibiting Notch signalling in the mouse intestine favours goblet cell differentiation in the proliferating cells of intestinal adenomas. Together with findings presented in this study, this suggests that both forward and reverse Notch signalling interact to define goblet cell differentiation in proliferating intestinal epithelium. The available data do not shed further light on the precise roles of Dll4 and its Notch receptors in the differentiation of goblet cells. Nevertheless, it is conceivable that manipulating Dll4–Notch signalling may have therapeutic relevance to colon cancer progression.

Activity of the hypoxia-inducible transcription factor isoforms, HIF-1 and HIF-2, is controlled by turnover of their *α*-subunits, HIF-1*α* and HIF-2*α*, respectively (Appelhoff *et al*, 2004). The data show that both HIF-1*α* and HIF-2*α* are expressed at similar frequencies in colon cancer. However, it was HIF-2*α* that significantly associated with VEGF expression, suggesting that the hypoxic induction of VEGF is predominantly controlled by HIF-2*α* in colon cancer. This is consistent with data by Mizukami *et al* (2004) who suggest that VEGF is induced in hypoxic regions of colorectal cancer by an HIF-1*α*-independent mechanism. Indeed, data from Knowles *et al* (2003) suggest that the availability of factors, such as oxygen, ascorbate and iron, may regulate which HIF isoform is active, even when both HIF-1 and HIF-2 are expressed. Therefore, it is possible that the microenvironment of colorectal cancer favours the activity of HIF-2 over HIF-1. Carbonic anhydrase 9 is predominantly regulated by HIF-1 (not HIF-2) activity (Sowter *et al*, 2003), but does not significantly associate with HIF-1*α* expression in colon cancer. This may be due to the confounding effects of the reported HIF-1-independent induction of CA9 by hypoxia (Kaluz *et al*, 2002). The positive association between endothelial Dll4 and epithelial VEGF in colon cancer, observed in this study, is also consistent with data from model systems in which Dll4 expression by endothelial cells is reported to be regulated by VEGF (Patel *et al*, 2005). Together, these data suggest that, in colon cancer, VEGF expression may be regulated by HIF-2 activity and the expression of VEGF may induce Dll4 expression in adjacent endothelial cells. This is consistent with data reported by Skuli *et al* (2009) who show that the deletion of HIF-2*α* in immortalised endothelial cells results in the downregulation of Dll4 expression.

The activity of HIF-1 and HIF-2 is reported to be regulated by hydroxylation of their *α*-subunits by the PHD family members (Appelhoff *et al*, 2004). The hydroxylation is believed to target the HIF-1 and HIF-2 *α*-subunits for destruction by the Von Hippel–Lindau tumour suppressor protein (Appelhoff *et al*, 2004). In this study the expression of the PHD proteins was strongly associated with each other, consistent with data in prostate cancer (Boddy *et al*, 2005), and suggesting that they are regulated by a common mechanism. Expression of the PHD proteins did not significantly associate with HIF-1*α*, consistent with studies in prostate cancer (Boddy *et al*, 2005), but were also unexpectedly positively associated with HIF-2*α*. The reasons for this are not clear, but suggest that the role of the PHD proteins under model physiological conditions may be different to their role in cancer. However, PHD2 and PHD3 are targets of HIFs and are induced by hypoxia (Appelhoff *et al*, 2004), which may explain the paradoxical association, which limits the HIF effects in a well-recognised feedback loop (D’Angelo *et al*, 2003). Alternatively, other factors may regulate HIF-2*α* expression in colon cancer, such as iron-deficiency anemia (Sanchez *et al*, 2007), confounding the data. Further work is needed to analyse the role of the PHD proteins in regulating HIF-1 and HIF-2 activity in human neoplasia.

In survival analyses, the absence of an observed prognostic association for HIF-1*α* in colon cancer is consistent with previous reports (Yoshimura *et al*, 2004). Moreover, the lack of a prognostic association for endothelial Dll4, a VEGF target (Patel *et al*, 2005), is in agreement with the lack of a prognostic association for VEGF in colon cancer in this study and elsewhere (Nanni *et al*, 2002; Zheng *et al*, 2003; Jubb *et al*, 2006a). In this study the expression of CA9 was not prognostic in terms of overall survival, in contrast to a report that expression of CA9 is a poor prognostic factor in colorectal cancer (Cleven *et al*, 2007). However, the series described by Cleven *et al* (2007) includes rectal cancers that have received neoadjuvant radiotherapy, which may confound their results as hypoxic cells are relatively resistant to radiation-induced cell death (Wouters and Brown, 1997). Unlike other published studies on hypoxia in colorectal cancer, no patients in this series of colon cancers received neoadjuvant therapy. Therefore, treatment effects do not confound the data presented in this study.

The association between HIF-2*α* and prognosis in colon cancer is consistent with previously published reports in two series of colorectal cancers (Yoshimura *et al*, 2004; Cleven *et al*, 2007), although our paper is the first to report the effect of HIF-2*α* in a large series of large intestinal cancers that have not received radiotherapy. Mathematical modelling suggests that hypoxic microenvironments provide evolutionary pressure, clonally selecting more aggressive neoplastic cells that are able to survive hypoxic stress (Anderson *et al*, 2006). Moreover, molecular markers of hypoxia have been associated with radiation treatment failure in rectal cancer (Korkeila *et al*, 2009). Together, these data suggest that cancers expressing HIF-2*α* are more aggressive and may warrant more intensive treatment.

This study has two principal limitations. First, it is exploratory, and although the results largely agree with the literature, they have not been internally validated. Limitations of sample size mean that the study is not adequately powered to detect small but statistically significant differences in survival between subsets. However, such differences are arguably too small to be clinically significant. Second, tissue microarrays are not always suitable for assessing markers of hypoxia and angiogenesis. Nevertheless, provided the cohort is large, there is sufficient core redundancy and the prevalence of positive markers is sufficiently high (as is presented in this study), any effects on the data should be minimised.

In summary, Dll4 is expressed in cancer-associated endothelial cells, but not the endothelium adjacent normal colonic mucosa. This study suggests that tumour endothelial expression of Dll4 may not be a significant prognostic factor, but is significantly associated with VEGF expression. In addition, Dll4 is expressed in some neoplastic cells with goblet cell differentiation, suggesting that Notch signalling can have a more direct role in colon cancer oncogenesis. Assessing which patients express Dll4 in their tumour endothelium may be critical in predicting response to new therapies that target Notch or VEGF signalling. Furthermore, the antibody may be useful for imaging the vasculature of tumours before therapy.

## Figures and Tables

**Table 1:** Statistical significance of associations between molecular variables

**Variable**		**HIF-2*α***	**CA9**	**VEGF**	**Dll4 Endo.**	**PHD1**	**PHD2**	**PHD3**
HIF-1*α*	*χ* ^2^	<0.01	3.43	0.96	2.08	0.23	<0.01	<0.01
	*P*	1.00	0.09	0.33	0.11	0.63	0.97	1.00
HIF-2*α*	*χ* ^2^	—	<0.01	18.78	4.5	4.24	10.41	11.75
	*P*		0.96	<0.0001	0.03^*^^,a^	0.04^*^	0.001	<0.001
CA9	*χ* ^2^	—		<0.01	<0.01	1.45	4.53	1.11
	*P*			1.00	1.00	0.23	0.03^*^	0.29
VEGF	*χ* ^2^	—	—	—	5.98	34.15	25.33	29.92
	*P*				0.01	<0.0001	<0.0001	<0.0001
Dll4 Endo.	*χ* ^2^	—	—	—	—	5.43	0.26	7.14
	*P*					0.01	0.61	0.008
PHD1	*χ* ^2^	—	—	—	—	—	16.04	32.90
	*P*						<0.0001	<0.0001
PHD2	*χ* ^2^	—	—	—	—	—	—	13.85
	*P*							0.0002

a Negative association.

All associations are positive unless otherwise stated.

^*^These *P-*values are not significant after correction for multiple testing by the Benjamini and Hochberg false discovery rate controlling procedure with a cutoff=0.05.

Abbreviations: Endo.=endothelial; PHD1=prolyl hydroxylase; VEGF=vascular endothelial growth factor; CA9=carbonic anhydrase 9; HIF=hypoxia-inducible factor; Dll4=delta-like ligand 4.

**Table 2:** Statistical significance of associations between molecular and categorical clinical variables

**Variable**		**pT**	**pN**	**M**	**LI**	**VI**	**Grade**	**Sex**	**Site**	**AC**
HIF-1*α*	*χ* ^2^	1.37	2.97	0.25	<0.01	1.37	6.26	0.18	5.55	1.10
	*P*	0.71	0.40	0.62	0.94	0.24	0.04^*^	0.67	0.02^*^	0.29
HIF-2*α*	*χ* ^2^	1.08	0.37	2.87	0.24	0.49	1.79	0.03	3.64	0.45
	*P*	0.78	0.95	0.09	0.62	0.49	0.41	0.88	0.06	0.50
CA9	*χ* ^2^	2.39	4.22	0.12	0.90	0.76	2.25	0.89	4.09	0.00
	*P*	0.50	0.24	0.73	0.34	0.38	0.33	0.27	0.04^*^	1.00
VEGF	*χ* ^2^	2.00	7.77	0.89	0.10	0.49	1.90	0.82	4.07	0.00
	*P*	0.57	0.05	0.35	0.75	0.49	0.39	0.37	0.04^*^	1.00
Dll4 Endo.	*χ* ^2^	0.40	1.77	<0.01	0.13	0.07	0.44	0.92	0.06	0.05
	*P*	0.94	0.62	1.00	0.72	0.79	0.80	0.34	0.81	0.83
PHD1	*χ* ^2^	9.18	3.28	0.13	0.30	0.26	9.61	0.25	<0.01	0.00
	*P*	0.03^*^	0.35	0.72	0.58	0.61	0.01^*^	0.62	0.93	1.00
PHD2	*χ* ^2^	1.83	0.99	2.28	1.09	0.11	1.05	0.12	3.80	0.00
	*P*	0.61	0.80	0.13	0.30	0.74	0.59	0.91	0.05	1.00
PHD3	*χ* ^2^	2.30	3.31	<0.01	0.60	1.43	5.99	1.43	0.80	0.09
	*P*	0.51	0.35	0.98	0.44	0.23	0.05	0.23	0.78	0.77

Abbreviations: AC=adjuvant chemotherapy; Endo.=endothelial; LI=lymphatic invasion; VI=vascular invasion; PHD=prolyl hydroxylase; VEGF=vascular endothelial growth factor; CA9=carbonic anhydrase 9; HIF=hypoxia-inducible factor; Dll4=delta-like ligand 4.

^*^These *P-*values are not significant after correction for multiple testing by the Benjamini and Hochberg false discovery rate controlling procedure 40 with a cutoff=0.05.

**Table 3:** Univariate analysis of overall survival

**Variable**	***N***	**Events**	**Median survival (months)**	**95% Confidence interval (months)**	***P-*value^a^**
Overall	177	102	83.3	58.9–107.7	
*HIF-1α*					
Negative	95	56	79.6	45.5–113.7	
Positive	60	38	77.6	40.8–114.4	0.91
					
*HIF-2α*					
Negative	74	37	101.5	81.5–120.7	
Positive	85	55	57.4	36.1–78.7	0.027
					
*CA9*					
Negative	55	32	79.6	30.2–128.9	
Positive	110	66	79.1	48.1–110.1	0.87
					
*VEGF*					
Negative	20	10	83.3	72.6–94.0	
Positive	149	88	79.6	49.2–110.0	0.46
					
*Dll4 Endo.*					
Negative	50	29	64.0	9.0–118.0	
Positive	125	72	89.0	64.2–113.6	0.94
					
*PHD1*					
Negative	46	26	79.1	46.0–112.2	
Positive	116	69	88.9	56.2–121.6	0.71
					
*PHD2*					
Negative	29	13	111.5	NR	
Positive	135	83	66.7	37.7–95.7	0.08
					
*PHD3*					
Negative	52	29	97.8	68.2–127.4	
Positive	120	70	68.9	39.9–97.8	0.67
					
*pT stage*					
1	5	2	NR		
2	13	4	NR		
3	77	37	110.9	97.9–124.0	
4	82	59	45.0	32.4–57.6	0.0003
					
*pN stage*					
0	103	45	139.9	98.1–181.7	
1	43	31	49.2	25.6–72.7	
2	28	24	18.1	1.8–34.3	
3	3	2	21.1	0–43.8	<0.0001
					
*M stage*					
0	156	82	100.0	82.1–118.0	
1	21	20	9.2	5.1–13.3	<0.0001
					
*LI*					
Absent	150	84	91.9	71.2–112.6	
Present	22	16	18.0	3.6–32.5	0.007
					
*VI*					
Absent	131	70	97.6	73.6–121.7	
Present	41	30	23.1	0–52.4	0.002
					
*Grade*					
Good	46	22	111.0	94.9–127.1	
Moderate	101	64	64.0	34.1–93.9	
Poor	11	8	28.9	0–83.5	0.028
					
*Sex*					
Female	80	45	88.9	61.6–116.2	
Male	97	57	79.6	40.3–118.8	0.68
					
*Site*					
Distal	73	45	77.6	41.7–113.5	
Proximal	104	57	88.9	57.7–120.0	0.50
					
*AC*					
No	110	56	101.1	82.5–119.7	
Yes	67	46	54.4	41.0–67.8	0.003

Abbreviations: AC=adjuvant chemotherapy; Endo.=endothelial; LI=lymphatic invasion; NR=not reached; VI=vascular invasion; PHD=prolyl hydroxylase; VEGF=vascular endothelial growth factor; CA9=carbonic anhydrase 9; HIF=hypoxia-inducible factor; Dll4=delta-like ligand 4.

a Log-rank test (Mantel–Cox).

**Table 4:** Multivariate analysis of overall survival

**Variable**	**Hazard ratio**	**95% Confidence interval**	**Association with shorter survival**	***P-*value^a^**
HIF-2*α*	1.61	1.01–2.57	Positive	0.04
pT stage	1.46	1.00–2.13	High pT	<0.05
pN stage	1.60	1.13–2.26	High pN	0.009
M stage	5.19	2.65–10.14	Present	<0.0001
LI	1.42	0.72–2.81		0.32
VI	1.23	0.69–2.20		0.48
Grade	1.14	0.73–1.80		0.56
AC	1.17	0.68–2.02		0.56

Abbreviations: AC=adjuvant chemotherapy; LI=lymphatic invasion; VI=vascular invasion; HIF=hypoxia-inducible factor.

a Cox regression.
