# Supplementary figures and images for: Expression of delta-like ligand 4 (Dll4) and markers of hypoxia in colon cancer
Source: Br J Cancer. 2009 Oct 20;101(10):1749–57. doi: 10.1038/sj.bjc.6605368 (PMC2778546; doi:10.1038/sj.bjc.6605368)

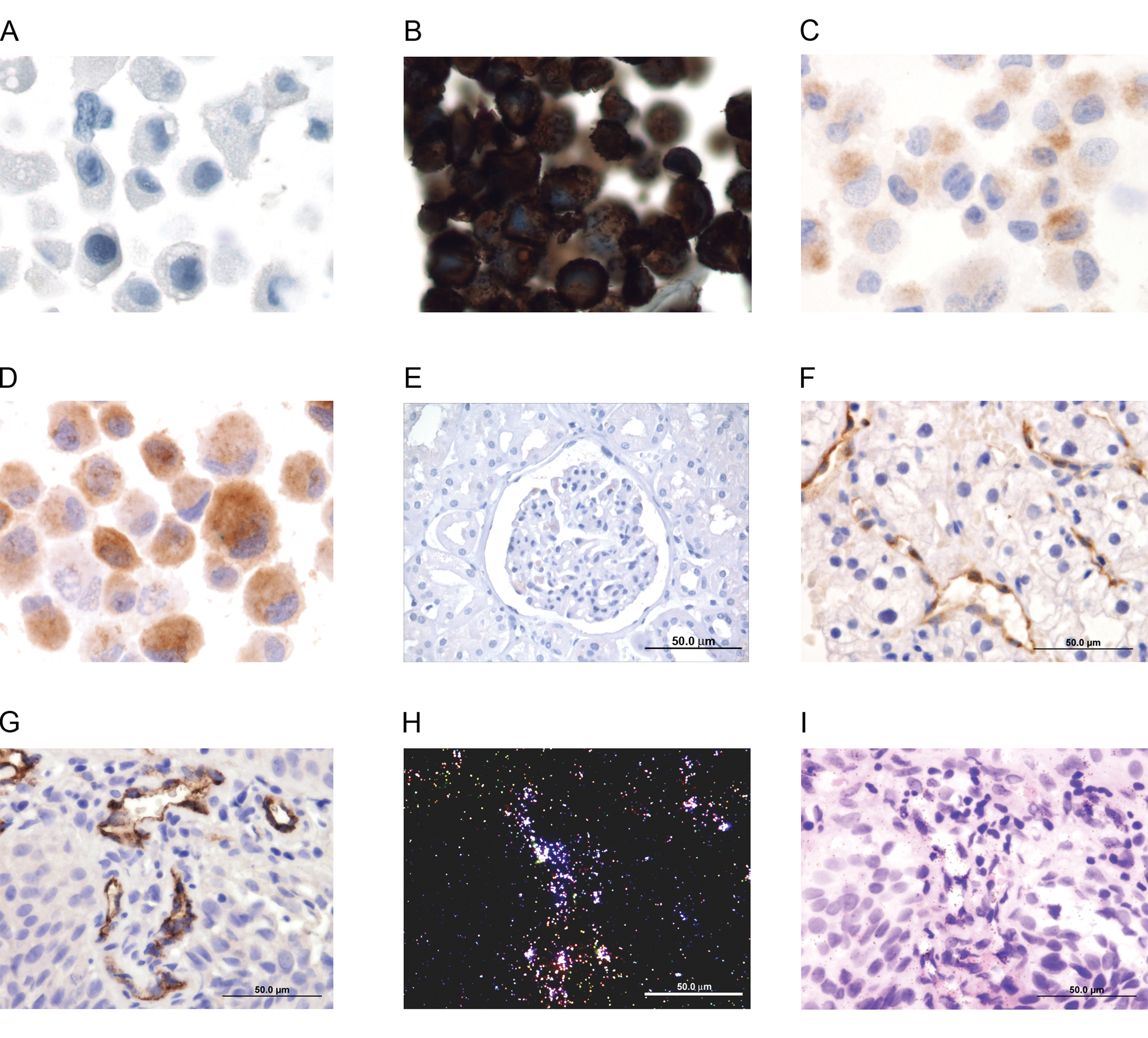

Supplement: Supplementary Figure 1 [file 6605368x1.tif]

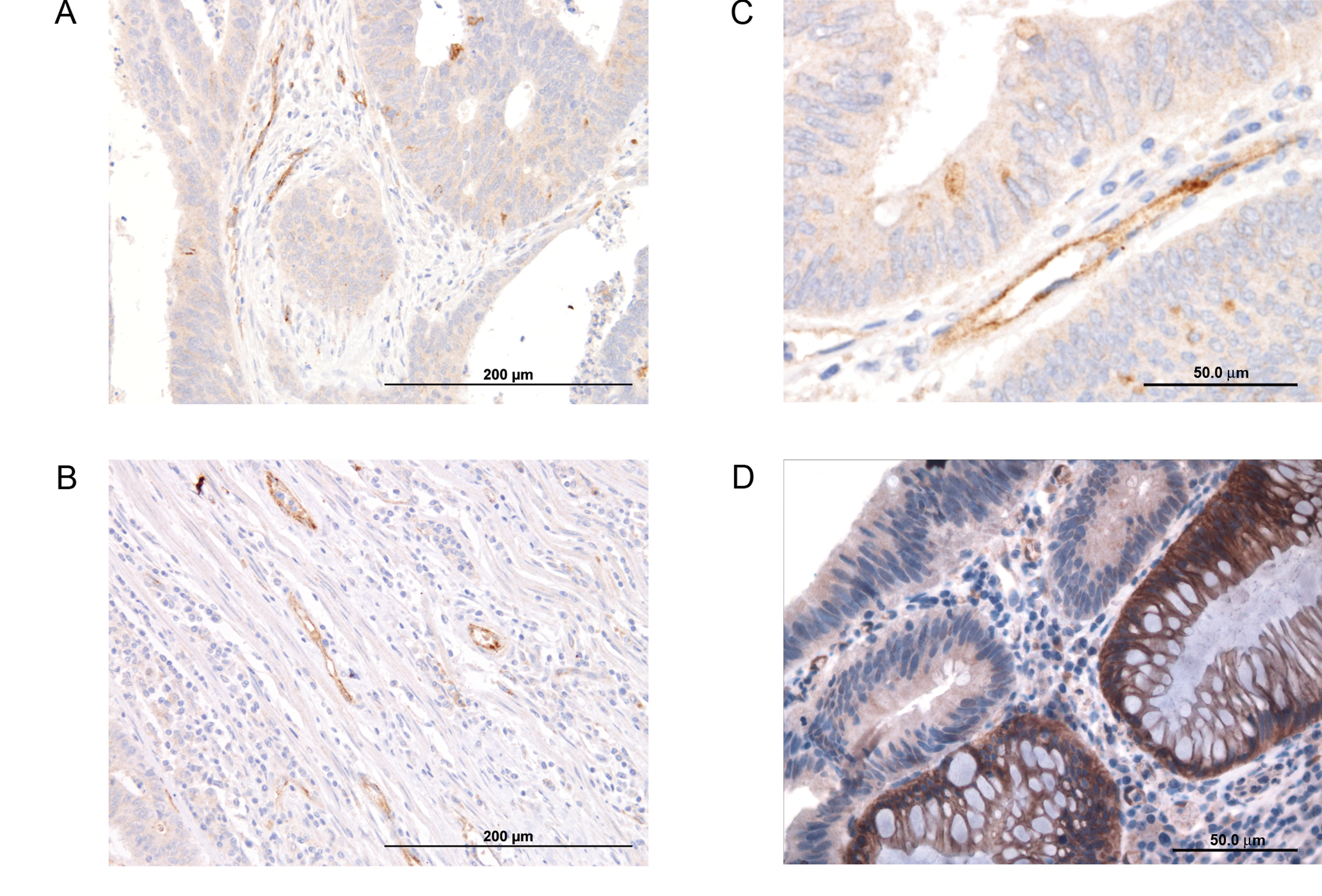

Supplement: Supplementary Figure 2 [file 6605368x2.tif]

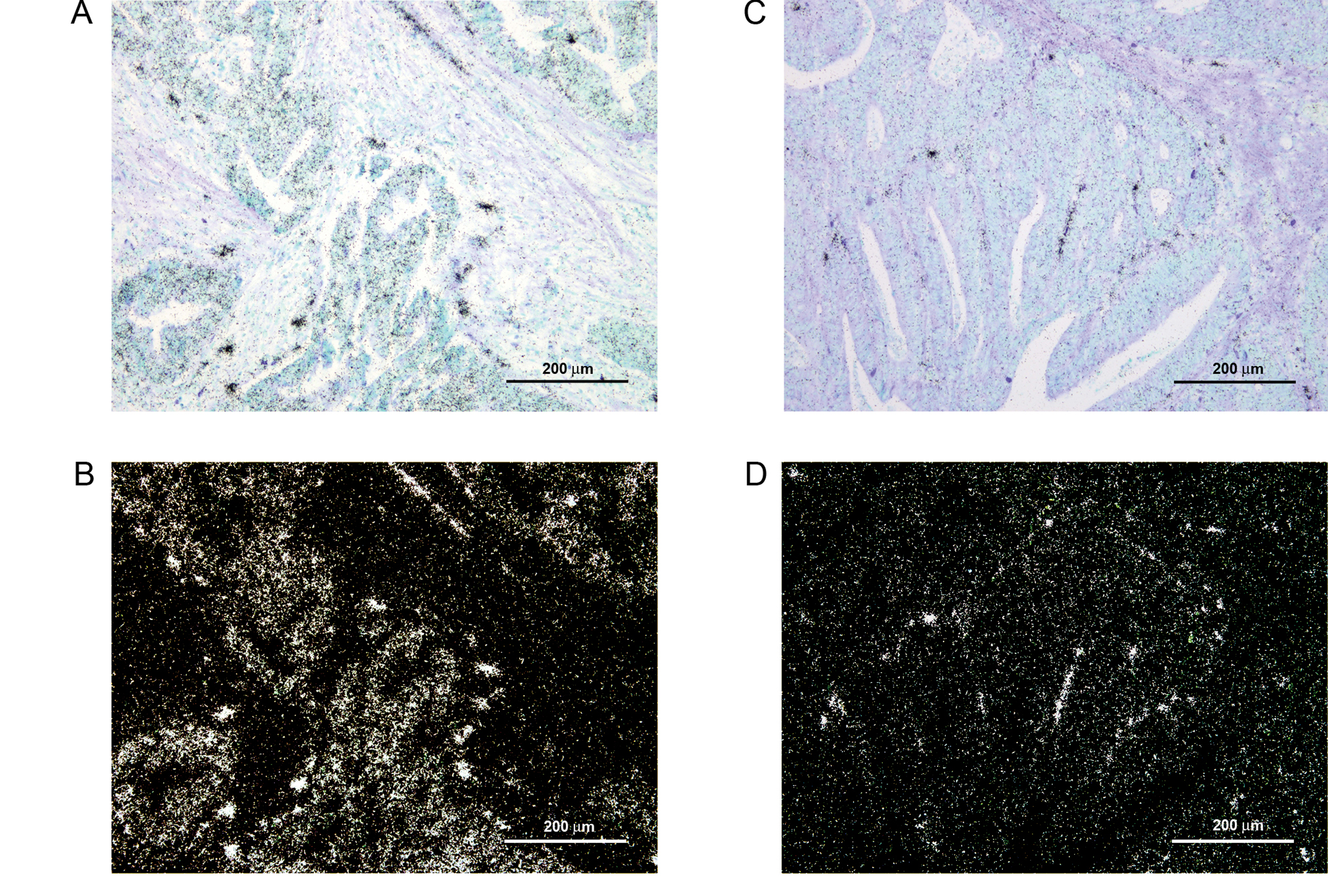

Supplement: Supplementary Figure 3 [file 6605368x3.tif]
