# Supplementary material for: Expression of delta-like ligand 4 (Dll4) and markers of hypoxia in colon cancer
Source: Br J Cancer. 2009 Oct 20;101(10):1749–57. doi: 10.1038/sj.bjc.6605368 (PMC2778546; doi:10.1038/sj.bjc.6605368)
Supplement: Supplementary Figure Legends [file 6605368x4.doc]

**Supplementary Figure 1:** Validation of anti-Dll4 monoclonal antibody, clone 242 for immunohistochemistry on formalin-fixed paraffin-embedded (FFPE) tissue. Immunohistochemistry for Dll4 on U87 cells transiently transfected with empty vector (A, 40x) or vector containing recombinant human Dll4 (B, 40x). Human umbilical vein endothelial cells showed immunoreactivity for Dll4 (C, 40x) that was increased when cells were treated with vascular endothelial growth factor (D, 40x). Normal kidney did not express Dll4 (E), but renal cell carcinoma did (F), in agreement with published studies. Bladder cancer showed co-localization of Dll4 expression by immunohistochemistry (G) and in situ hybridization (H, dark-field and I, bright-field) in serial sections.

**Supplementary Figure 2:** Additional immunohistochemistry for Dll4 showing endothelial expression in a colonic adenocarcinoma (A-C) and epithelial expression associated with goblet cell morphology in a neoplastic adenomatous crypt (D).

**Supplementary Figure 3:** In situ hybridization for Dll4 in whole sections of colon cancer, exemplifying a tumour with endothelial and epithelial expression of Dll4 (A, bright-field and B, dark-field) and a tumour with only endothelial expression of Dll4 (C, bright-field and D, dark-field).
